# Development and validation of the African Women Awareness of CANcer (AWACAN) tool for breast and cervical cancer

**DOI:** 10.1371/journal.pone.0220545

**Published:** 2019-08-06

**Authors:** J. Moodley, S. E. Scott, A. D. Mwaka, D. Constant, J. N. Githaiga, T. S. Stewart, A. Payne, L. Cairncross, N. I. M. Somdyala, F. M. Walter

**Affiliations:** 1 Women’s Health Research Unit, School of Public Health and Family Medicine, Faculty of Health Sciences, University of Cape Town; Observatory, Cape Town, South Africa; 2 Cancer Research Initiative, Faculty of Health Sciences, University of Cape Town; Observatory, Cape Town, South Africa; 3 SAMRC Gynaecology Cancer Research Centre, Faculty of Health Sciences, University of Cape Town, Observatory, Cape Town, South Africa; 4 Centre for Oral, Clinical and Translational Sciences, Faculty of Dentistry, Oral and Craniofacial Sciences, King’s College London, London, United Kingdom; 5 Department of Medicine, School of Medicine, College of Health Sciences, Makerere University, Kampala, Uganda; 6 Department of Obstetrics & Gynaecology, Plymouth Hospitals NHS Trust, Derriford Hospital, Plymouth, Devon, United Kingdom; 7 Surgical Endocrine Oncology Unit, Division of General Surgery, Department of Surgery, University of Cape Town; Observatory, Cape Town, South Africa; 8 Eastern Cape Cancer Registry, South African Medical Research Council, Burden of Disease Research Unit, Tygerberg, South Africa; 9 The Primary Care Unit, Department of Public Health & Primary Care, University of Cambridge, Cambridge, United Kingdom; Mayo Clinic College of Medicine, UNITED STATES

## Abstract

**Background:**

Measuring factors influencing time to presentation is important in developing and evaluating interventions to promote timely cancer diagnosis, yet there is a lack of validated, culturally relevant measurement tools. This study aimed to develop and validate the African Women Awareness of CANcer (AWACAN) tool to measure awareness of breast and cervical cancer in Sub-Saharan Africa (SSA).

**Methods:**

Development of the AWACAN tool followed 4 steps: 1) Item generation based on existing measures and relevant literature. 2) Refinement of items via assessment of content and face validity using cancer experts’ ratings and think aloud interviews with community participants in Uganda and South Africa. 3) Administration of the tool to community participants, university staff and cancer experts for assessment of validity using test-retest reliability (using Intra-Class Correlation (ICC) and adjusted Kappa coefficients), construct validity (comparing expert and community participant responses using t-tests) and internal reliability (using the Kuder-Richarson (KR-20) coefficient). 4) Translation of the final AWACAN tool into isiXhosa and Acholi.

**Results:**

ICC scores indicated good test-retest reliability (≥ 0.7) for all breast cancer knowledge domains and cervical cancer risk factor and lay belief domains. Experts had higher knowledge of breast cancer risk factors (p < 0.001), and cervical cancer risk factors (p = 0.003) and symptoms (p = 0.001) than community participants, but similar knowledge of breast cancer symptoms (p = 0.066). Internal reliability for breast cancer risk factors, lay beliefs and symptom and cervical cancer symptom subscales was good with KR-20 values > 0.7, and lower (0.6) for the cervical cancer risk subscale.

**Conclusion:**

The final AWACAN tool includes items on socio-demographic details; breast and cervical cancer symptom awareness, risk factor awareness, lay beliefs, anticipated help-seeking behaviour; and barriers to seeking care. The tools showed evidence of content, face, construct and internal validity and test-retrest reliability and are available for use in SSA in three languages.

## Introduction

Breast and cervical cancer are the leading causes of cancer morbidity and mortality in women in Sub-Saharan Africa (SSA). Breast cancer is the most commonly diagnosed cancer among women in SSA, whilst cervical cancer remains the leading cause of cancer death [1]. Most SSA countries do not have cervical or breast cancer screening programs and the majority of cancers (85–90%) present symptomatically and at an advanced stage [2].

Studies have shown that for symptomatic breast cancer, a shorter time between recognition of symptoms by women and first medical consultation is associated with early stage disease, and better breast cancer survival [3–6]. Further, a substantial proportion of advanced stage diagnosis of poorly differentiated breast cancer cases could be avoided if patients had presented within one month of detecting symptoms [4]. For both breast and cervical cancer there are often subtle but important symptoms in early stage disease, yet women may misinterpret these symptoms or wait until symptoms (and disease) progress before they seek medical attention [7–10]. Research outside SSA has indicated that cancer symptom awareness among the general population can be low [11–12], and that interventions to increase awareness lead to better outcomes. For example, in Malaysia, raising public awareness of symptoms has been associated with earler diagnosis of cervical cancer [13].

For people with potential symptoms of cancer, the pathway to cancer diagnosis is complex and influenced by many factors such as symptom and risk awareness, beliefs and barriers to accessing care [9,10,14–18]. Measuring these factors is important in developing and evaluating interventions to promote timely cancer diagnosis. A recent scoping study highlighted the need for local research arising from low-and middle-income countries to adequately address breast and cervical cancer prevention and control [19].

Although a few studies have reported on breast and cervical cancer symptom awareness, risk perception and help-seeking behavior in African settings [9,10,20,21], measurement and comparison is hampered by the lack of validated, culturally relevant measurement tools. The Cancer Awareness Measure (CAM) and the Awareness and Beliefs about Cancer (ABC) tools, developed and validated in the UK to reliably assess cancer awareness in the general public, are questionnaires that include generic and cancer site specific modules [22–26]. The CAM and ABC tools could be of value beyond the UK, but as they were developed in a very different cultural setting they need to be adapted and validated for use in SSA. A Kenyan research team adapted the Breast Cancer Awareness Measurement (BCAM) for local use and reported on validity testing of two of the tool domains (symptom awareness and barriers to screening) [27]. The team reported good content validity and internal reliability for the two domains, demonstrating that the BCAM could be adapted for use in African populations, but they also recommended further validation. The aim of the current study was to develop and validate an African Women’s Awareness of CANcer (AWACAN) tool to measure awareness of symptoms and risk factor for breast and cervical cancer, lay beliefs, confidence in appraising potential symptoms, help-seeking behavior and barriers to health care.

## Methods

The four step processes of 1) generating tool items, 2) refining and 3) validating the English version of the AWACAN tool and, 4) developing local language versions of the tool are described below. The outcomes of Step 3 are presented in the results section. Ethics approval for the study was obtained from University of Cape Town, Faculty of Health Sciences Human Research Ethics Committee (HREC 544/2016), the Lacor Hospital Institutional Research Ethics Committee (LHIREC 027/11/2016) and the Ugandan National Council of Science and Technology (UNCST 194/212/01). Written informed consent was obtained from all participants. Permission to access health facilities was obtained from all relevant local health authorities. Permission to conduct interviews with non-medical university staff was obtained from the institutional human resource department.

### Step 1: Generating items for the AWACAN tool

Items to assess breast and cervical cancer symptom and risk factor awareness and help-seeking behavior were generated by reviewing the UK (BCAM) and Cervical Cancer Awareness Measurement (CCAM) tools, the ABC tool, and studies conducted in Africa that used these or other tools to measure constructs of interest to our study [10, 25–27]. Items relating to overall cancer awareness (not specific to breast or cervical cancer) and awareness of screening programs were excluded as this was not the focus on our study. The multi-disciplinary research team reviewed the list of items, making changes so that the terminology would be applicable to the African setting. A first version of the AWACAN tool was drafted and consisted of a total of 131 items: 13 socio-demographic; 57 breast cancer; 42 cervical cancer and; 19 barriers to seeking health care questions. The list of variables included; having heard of or knowing someone with breast or cervical cancer; knowledge of age-related risk; knowledge of risk factors and symptoms of breast and cervical cancer (evidence-based); and risk lay beliefs such as exposure to an unhygienic environment, placing objects in undergarments and witchcraft. Based on experiences with the use of BCAM in Kenya [27], we chose to commission images of breast cancer signs on dark skinned women, developed by a graphic designer in consultation with the breast cancer surgeon on the team (LC). Items also assessed confidence in detecting breast or cervical cancer symptoms. Help-seeking behavior variables assessed whether participants would ignore symptoms, try self-medication, inform a friend/relative, visit a traditional healer and visit a medical facility. Anticipated time to presentation to medical facilities and traditional healers was measured on a scale from immediately to more than 1 year. Barriers to seeking care were measured in terms of emotional, practical and service related barriers. The AWACAN tool was designed to be delivered via a structured interview rather than self-completed questionnaire.

### Step 2: Refinement of items for the AWACAN tool

#### To assess content validity

A group of cancer experts based in SSA (n = 48) were invited to complete an anonymous online survey and score the AWACAN tool items in terms of clarity and relevance [(1(poor) - 4 (excellent)], provide reasons for any item scoring < 3, and identify missing items. The experts included breast or cervical cancer health professionals, epidemiologists and public health researchers and were identified from peer-reviewed literature, websites of relevant academic and research organizations and through contacts of research team members. A content validity index [(CVI) number of raters giving a score of ≥3/total number of raters] was calculated for each tool item [28]. Any item with a CVI >78% was regarded as having an adequate CVI as recommended by Lynn *et al*. [28]. In total 25 experts completed the online survey.

#### To assess face validity

Cognitive interviews using think-aloud methodology [29–30] were conducted with English-speaking women aged 18 years and older to assess interpretation and understanding of the tool items. Participants, excluding women with a history of breast or cervical cancer, were recruited from primary care clinics in urban primary health care clinics in South Africa (n = 10) and Uganda (n = 10) and asked to think aloud as they responded to the structured interview. Interviews were transcribed verbatim and analysed to identify areas of ambiguity and items causing distress.

Findings from both the cancer expert review and the cognitive interviews were reviewed and tool items revised where necessary. This resulted in: items being deleted; questions being reworded/rephrased for clarity; definitions being added for poorly understood terms and; a revision of response options for some questions. Items on age-related risk factors (e.g. Do you think that a woman younger than you is more likely to get breast cancer?) were deleted as they were poorly understood by participants and all had a low CVI. An item on participant’s religion was deleted as it had a low CVI (0.56) with cancer experts noting that it could be viewed as being judgemental. Five questions were identified as being redundant as they were covered elsewhere in the questionnaire and were removed. Explanations were added for the terms “hormone replacement therapy” and “menopause” as these were poorly understood by women. All questions with Likert response options had a low CVI (< 78%) and were poorly understood by women during the cognitive interviews. Options for these questions were revised. For example, the question “How confident are you that you would notice a change in your breast?” with response options a. very confident b. fairly confident c. slightly confident and d. not confident at all was changed to “Are you confident that you would notice a change in your breast?” with response options a. yes b. no c. not sure.

The refinement process resulted in a second version of the AWACAN tool with a total of 123 items: 12 socio-demographic questions; 53 breast cancer symptom,risk factor, confidence and help-seeking questions; 39 cervical cancer symptom, risk factor, confidence and help-seeking questions and 19 items on barriers to seeking care.

### Step 3: Validation of the AWACAN tool

#### Participants and procedure

Three groups of participants completed the second version of the AWACAN tool:

- Community participants: Women aged ≥ 18 years and able to speak and understand English were recruited from primary care clinics at the sites used for the cognitive interviews described above. English proficiency was assessed by a trained interviewer during the process of providing study information and obtaining informed consent. Participants that were unable to understand and speak English were excluded. Women were stratified by age during recruitment to ensure representation by older (≥ 50 years) and younger (< 50 years) participants. Trained field workers administered the tool to participants.
- Cancer experts: Experts who participated in the content validaty exercise (Step 2A) were invited to complete an on-line version of the tool.
- A convenience sample of non-medical university staff: aged 18 to 65 years were recruited from the University of Cape Town. A trained interviewer administered the tool on two occasions, two weeks apart. Participants were stratified by age (≥ 50 years and < 50 years).

#### Data management

All data was entered into Qualtrics and analysed using STATA (Version 13.0). Scores for risk factor knowledge and symptom awareness were calculated with each correct answer to the evidence-based questions in the closed/prompted sections scoring 1 point. Lay beliefsthat were correctly identified as not being a risk factor scored 1 point.

The revised 123-item tool was assessed in terms of indicators of test-retest reliability, construct validity and internal reliability.

#### Test-rest reliability analysis

For categorical variables related to awareness (having heard of or knowing someone with relevant cancer), help-seeking behaviour and confidence test-retest reliability was calculated using the Brennan and Prediger prevalence-adjusted and bias-adjusted Kappa. Adjusted Kappas were calculated as the unadjusted Cohen Kappa is prevalence dependant and misleading when ratings fall into a single category [31,32]. We present both adjusted Kappa and Cohen’s Kappa values, as adjusted Kappa presented alone can be difficult to interpret. Kappa coefficients were interpreted as follows: <0.00 as poor; 0.00–0.20 as slight; 0.21–0.40 as fair; 0.41–0.60 as moderate; 0.61–0.80 as substantial and 0.81–1.0 as almost perfect agreement [33].

For knowledge domains, test-retest reliability was assessed by comparing the mean test and retest scores of the non-medical staff, using the intra-class correlation coefficient (ICC) with values below 0.5 indicating poor reliability, between 0.5 and 0.75 moderate reliability, between 0.76 and 0.9 good reliability, and above 0.9 excellent reliability [34].

#### Construct validity analysis

Construct validity was assessed by comparing knowledge scores between the cancer experts and community participant group using t-tests. We hypothesized that if the tool had construct validity then cancer experts would have significantly (p<0.05) higher awareness compared to community respondents.

#### Internal reliability analysis

Internal reliability for the risk factor and symptom knowledge constructs were assessed using the Kuder-Richarson (KR-20) coefficient of reliability and the item-to-total correlation coefficients. A KR-20 of > 0.7 was considered to indicate good internal reliability [35,36]. For the item-to-total correlation, a value greater than 0.2 was an indicator that an item was related to the overall scale [22,37].

#### Sample size

Typically, studies measuring internal reliability are based on the assumption that the number of respondents should exceed the number of items by 2 to 20 subjects per item, with a minimum of 100 subjects to ensure stability of the variance-covariance matrix [38]. Our sample size was based on an initial anticipated 60-item per cancer tool. Applying a 3:1 subject to item ratio we aimed to recruit a total of 180 to ensure a reasonably precise estimate the KR-20.

### Step 4: Development of the local language versions of the tool

The English version of the final AWACAN tool was translated into isiXhosa (the 2^nd^ commonest of 11 official languages in SA and Acholi (spoken in Northern Uganda and parts of Kenya and Tanzania) by Acholi and isiXhosa native speakers, and back translated into English to ensure comparability [39,40]. Forward and backward translations were also undertaken by a university-based language translation unit in South Africa and by health educators and senior clergy experienced in translating religious and government documents from English into Luo/Acholi. To ensure conceptual and cultural equivalence, cancer experts (SA = 4, Uganda = 4) fluent in both English and the local language scored tool items from 1(poor) to 4(excellent) on equivalence of meaning comparing the English and locally translated version. For items that were scored as 3 or less, experts were asked to suggest changes. Cognitive interviews using think-aloud methodology were carried out with local-language speaking community participants using the IsiXhosa version (SA, n = 8) and the Luo/Acholi version (Uganda, n = 10) to assess whether questions were understood as intended. Participants were recruited from primary health care clinics at urban and rural sites in South Africa and Uganda. Interviews were conducted by trained local interviewers conversant in the local language.

This process resulted in minor changes; these included correction of spelling errors, changes in phrasing to language used more commonly in communities, and inclusion of words that had been inadvertently omitted in the translation.

## Results

In total there were 139 community (SA n = 72, Uganda n = 67), 23 non-medical staff and 20 cancer expert participants who completed the validation studies (Step 3). Table 1 outlines the socio-demographic characteristics of the participant groups.

**Table 1:** Socio-demographic characteristics of participants.

	Community SA N = 72	Community Uganda N = 67	Non-medical staff N = 23	Cancer experts N = 20
**Median age in years (IQR)**	40.5 (30–53)	40.0 (21–73)	52.0 (41–55)	49.0 (41–54)
**Highest educational level completed**				
No formal schooling	1 (1.4%)	0 (0.0%)	0 (0.0%)	0 (0.0%)
Primary	8 (11.1%)	17 (25.4%)	0 (0.0%)	0 (0.0%)
Secondary	57 (79.2%)	50 (74.6%)	4 (17.4%)	0 (0.0%)
More than secondary	6 (8.3%)	0 (0.0%)	19 (82.6%)	19 (100.0%)*
**Employed**				
Yes	29 (40.3%)	21 (31.3%)	23 (100.0%)	20 (100.0%)
**Current relationship status**				
Married/Living with a partner	30 (41.7%)	30 (44.8%)	13 (56.5%)	15 (75.0%)
Single	33 (45.8%)	4 (6.0%)	8 (34.8%)	4 (21.1%)
Separated/Divorced	6 (8.3%)	12 (17.9%)	2 (8.7%)	0 (0.0%)
Widowed	3 (4.2%)	19 (28.4%)	0 (0.0%)	0 (0.0%)
Did not answer	0 (0.0%)	2 (3.0%)	0 (0.0%)	1 (5.0%)

SA: South Africa

IQR: interquartile range

* 1 missing record

### Test-retest reliability

There was no loss-to follow up between the first and second round of interviews. The percentage of agreement for responses to the socio-demographic questions was high (> 96% for all questions and total agreement for 8 of the 11 socio-demographic variables). Kappa scores for the socio-demographic questions ranged between 0.83 and 1.00.

Table 2 shows the agreement, the Brennan and Prediger prevalence-adjusted and bias-adjusted kappa and the unadjusted kappa scores in brackets for items relating to awareness, help-seeking behavior and confidence. For breast cancer, the adjusted kappa co-efficient ranged from 0.57 to 1.0 with one item (‘self-medicate’) showing moderate agreement and other items indicating substantial or almost perfect agreement. For cervical cancer, the adusted kappa coeeficients ranged from 0.65–1.0 i.e. all showed substantial or higher agreement. Unadjusted kappa co-efficient (shown in brackets) ranged from -0.06 to 1.0 for both cancers.

**Table 2:** Test-retest reliability of awareness, help-seeking behavior and confidence.

	Breast cancer		Cervical cancer	
	% Agreement	Adjusted Kappa (Unadjusted Kappa)	% Agreement	Adjusted Kappa (Unadjusted Kappa)
Heard of/know someone with breast/cervical cancer				
Ever heard of breast/cervical cancer	100	N/A–all yes	100	N/A all yes
Know someone with breast/cervical cancer	95.7	0.91 (0.65)	95.7	0.91 (0.89)
Help-seeking behavior for breast/cervical symptom				
Ignore it	95.7	0.91 (0.00)	87.0	0.74 (-0.06)
Hope it would go away	73.9	0.61 (0.50)	82.6	0.65 (0.65)
Self-medicate	78.3	0.57 (0.16)	95.7	0.91 (0.88)
Tell someone close to me	100	1.0 (1.0)	87.0	0.80 (0.52)
Pray for healing	100	1.0 (1.0)	100	1.0 (1.0)
Visit a traditional healer	95.7	0.91 (0.65)	95.7	0.91 (0.65)
Visit a health care facility	87.0	0.74 (-0.06)	87.0	0.74 (-0.06)
Behaviour and confidence				
Ever check breasts	100	1.0 (1.0)	N/A	
Confidence in noticing breast/cervical change	87.0	0.74 (0.51)	82.6	0.74 (0.56)
Ever seen a healthcare practitioner for breast/cervical change	91.3	0.83 (0.83)	87.0	0.80 (0.68)
Ever seen a traditional healer for breast/cervical change	87.0	0.74 (0.74)	87.0	0.74 (0.69)

Test-retest reliability was moderate for knowledge of known risk factors, good for symptoms and excellent for lay beliefs of breast cancer (Table 3). For cervical cancer, test-retest reliability was moderate for known risk factors, lay beliefs and the symptom domain.

**Table 3:** Test-retest reliability of knowledge domains (N = 23).

	Breast cancer		Cervical cancer	
	Test-retest reliability ICC	p-value	Test-retest reliability ICC	p-value
Known risk factors	0.70	<0.001	0.71	<0.001
Risk lay beliefs	0.91	<0.001	0.75	<0.001
Symptoms	0.77	<0.001	0.50	0.001

ICC: intraclass correlation

### Construct validity

A high proportion of community participants had heard of breast and cervical cancer (90% and 84% respectively) and there were no statistically significant differences between experts and community members for these items (1-sided Fischer’s exact test p value = 0.184 and 0.222 respectively).

For breast cancer, community participant and expert scores were significantly different for knowledge of evidence-based risk factors and risk lay beliefs but similar for breast cancer symptoms (Table 4). There was clear discrimination between the community participants and cancer experts’ knowledge of evidence-based risk factors and symptoms of cervical cancer. Knowledge did not differ significantly for the 1 cervical cancer risk lay belief (p = 0.444).

**Table 4:** Comparison of expert and community participant knowledge.

	Breast Cancer				Cervical Cancer			
Knowledge domain		Community	Experts	p-value		Community	Experts	p-value
	Max. score	n	n		Max. score	n	n	
		Mean	Mean			Mean	Mean	
		sd	sd			sd	sd	
Known risk factors	13	124	17	<0.001^#^	10	116	15	0.003^#^
		5.2	11.1			6.7	8.3	
		2.8	2.1			1.9	1.8	
Risk lay beliefs	7	125	18	<0.001^#^	1	116	16	0.444^#^
		2.5	6.1			0.8	0.9	
		1.7	1.1			0.4	0.3	
Symptoms	15	139	18	0.066^#^	11	138	17	0.001^##^
		12.7	13.9			7.6	8.9	
		2.7	1.8			2.3	1.3	

Max.: Maximum

sd: standard deviation

^#^P values for T-test for groups with equal variances

^##^ P values for T-test for groups with unequal variances

### Internal reliability

The internal reliability for the breast cancer knowledge questions was good with KR-20 values > 0.7 for the known risk factors, lay belief and symptom subscales (Table 5). For cervical cancer the KR-20 for the known risk factors subscale was lower (0.60). Cervical cancer risk factors items with a very low item-to-total correlation were: HPV (0.18), using birth control/family planning pills for more than 5 years (0.13), not going for regular screening (0.09). Internal reliability for the cervical cancer symptom questions was good (0.80).

**Table 5:** Internal reliability of the AWACAN tool.

	Breast Cancer			Cervical cancer		
Knowledge domain	No. of items per domain	No. of responses	Kuder-Richarson coefficient of reliability	No. of items per domain	No. of responses	Kuder-Richarson coefficient of reliability
Known risk factors	13	164	0.78	10	154	0.60
Risk lay beliefs	7	166	0.73	1	154	N/A^#^
Symptoms	15	180	0.79	11	179	0.80

#N/A, not applicable as includes only 1 item.

### Final AWACAN tool

Following assessment of reliability and validity, further adjustments to phrasing of some items were made to improve clarity. The number of questions related to barriers to seeking care was reduced (7 questions removed) as interviewers noted that participants found that this section was too long. In addition the wording of questions was changed to better indicate that these were barriers personally experienced by the respondent. Responses to open questions on risk factors and symptoms were reviewed to identify additional lay beliefs.

The final version of the AWACAN tool has a total of 115 items:

- 12 socio-demographic questions;
- 50 breast cancer symptom, risk factor awareness, confidence and help-seeking measures
  - 2 introductory questions
  - 1 open question on risk factors
  - 13 closed prompted evidence-based risk factor questions
  - 6 closed prompted questions on lay beliefs related to risk (can serve as distractor items)
  - 1 open question on symptom awareness
  - 15 closed prompted questions on symptom awareness items. Images of symptoms as they would appear on dark-skinned women were added for 3 items: change in the position of the nipple, pilling in of the nipple, and dimpling of the breast skin
  - 1 closed prompted question on symptom lay belief (can serve as distractor item)
  - 7 items related to help-seeking behaviour
  - 4 items on confidence in relation to breast changes
- 41 cervical cancer symptom, risk factor awareness, confidence and help-seeking measures
  - 2 introductory questions
  - 1 open question on risk factors
  - 11 closed prompted evidence-based risk factor questions
  - 4 closed prompted questions on lay beliefs related to risk (can serve as distractor items)
  - 1 open question on symptom awareness
  - 11 closed prompted questions on symptom awareness items
  - 1 closed prompted question on symptom lay belief (can serve as distractor item)
  - 7 items related to help-seeking behaviour
  - 3 items on confidence in relation to detecting cervical cancer signs/symptom
- 12 items on barriers to seeking care for breast and cervical cancer

See S1 Appendix The AWACAN tool for breast and cervical cancer English version and S2 Appendix Images of breast cancer changes. The English, isiXhosa and Acholi versions of the tool as well as the breast images are available at www.awacan.online

## Discussion

To our knowledge this is the first validated tool measuring breast and cervical cancer symptom and risk factor awareness, lay beliefs, confidence in appraising potential symptoms, help seeking behaviour and barriers to care in SSA populations. The AWACAN tool follows a similar format to the BCAM and CCAM [25,26]. Knowledge of risk factors and symptoms are first assessed with open-ended questions followed by a prompted checklist of known factors. Participants are not permitted to revise open-ended responses. This sequence of questioning allows respondents an opportunity to describe their own views without the possibility of guessing or being limited by the pre-existing options of the prompted questions that follow. Although responses to open-ended questions are more time-consuming to analyze compared to prompted questions, they can provide insights into terms and phrases commonly used to describe risk and symptoms which could assist in providing culturally relevant and easily understood language for interventions. Open-ended responses can also assist in identifying locally relevant risk and symptom lay beliefs.

Our AWACAN tool performed well in terms of test-retest reliability indicating that the questions are stable over time. Most socio-demographic, help-seeking behaviour and confidence items achieving substantial or higher adjusted kappa coefficients and all knowledge sub-scales showing moderate or higher reliability as measured by the ICC.

The AWACAN tool discriminated well between the expert and community level knowledge of breast cancer risk factors and risk lay beliefs. However, breast symptom knowledge was similar between these two groups. This could be a result of greater public awareness of breast symptoms, which is a focus of many breast awareness campaigns. Knowledge of cervical cancer items risk factors and symptoms was significantly higher in cancer experts compared to community participants, establishing construct validity for these subscales. At the stage of psychometric testing we only had one cervical cancer risk lay belief and although experts were better able to identify this lay belief the difference was not statistically different. Using responses from the open-ended questions we were able to identify additional lay belief items.

Internal reliability was good for our breast cancer questions, with all knowledge domains achieving a KR-20 above the recommend cut-off value of 0.7. Internal reliability was also high (KR-20 = 0.8) for the cervical cancer symptom domain, but lower (KR-20 = 0.6) for the subsection on risk factors. The three risk factor items contributing to this low internal reliability (HPV, use of birth control/family planning pills, not going for regular screening) were retained in the final tool on grounds of content validity.

After establishing good validity for the English version of the tool, it was translated into two 2 local languages (isiXhosa and Acholi), taking into account conceptual and cultural equivalence. In addition to these translations, there are some other important differences between our validated AWACAN tool and the BCAM and CCAM tools. Our tool includes additional prompted items on symptom and risk lay beliefs that were identified from the literature [27], and in responses to the open questions administered during the AWACAN development and validation process. These questions provide insights into lay beliefs that may need to be addressed when developing SSA-relevant and targeted interventions to improve timely diagnosis in these populations. In addition, the lay belief questions which are interspersed between known risk factors and symptoms serve as distractor items. For effectiveness, distractor items should represent incorrect responses that study participants could be expected to produce and avoid obviously implausible responses [41]. We were able to identify a number of breast and cervical cancer risk lay beliefs, but only one breast and one cervical cancer symptom lay belief. When the AWACAN tool is used in future studies, evaluation of open responses could assist in identifying additional locally relevant symptom distractor items.

Based on the recommendation of Wachira *et al* [27], we developed images for 3 breast cancer symptoms on dark-skinned women to be used with the tool. We believe that this is an important addition to cancer awareness measurement tools in our context. Further, these images can be used in awareness raising interventions. The AWACAN tool also includes simple explanations for terms that were not well understood by participants in our setting (e.g. menopause). Compared to the CAM tools our final tool does not include age-related risk factors as these were poorly understood by community participants and also received a low CVI score from experts.

The BCAM and CCAM tools make use of Likert scales, which call for a graded response to a series of statements about risk and confidence. Likert scales are widely used in surveys, however studies have reported challenges such as difficulty in understanding graded response options, skipping questions or bias toward either the midpoint or extreme options [42,43]. During cognitive testing we noted that participants in both SA and Uganda had difficulty understanding the Likert-type scales used in the CAM tools. Questions were thus modified in our tool to allow for simpler response options.

Understanding women’s help-seeking behaviour and potential barriers to care are critical to informing efforts to improve timely diagnosis of these cancers. The ABC and CAM tools includes measures of barriers to care and help-seeking behaviour relevant in high-income setting [23,25,26]. We included additional questions to reflect the different realities in the SSA context. For example, traditional healers are often consulted for health-related issues in SSA [44] and are included in our measures. We reduced the number of questions on barriers to seeking care in the final tool and made substantial changes to the wording of the remaining questions. Validity of this domain of our questionnaire will be further evaluated in future use of the tool and attention paid to context-specific variations.

Over the past few decades, there has been an increasing recognition that a holistic and comprehensive approach to women’s health is central to sustainable development [45,46]. We developed the AWACAN tool so that the two most common cancer in females in SSA can be addressed in one study to maximise data collection from women. However the tool can also be used for either breast or cervical cancer assessments alone.

A limitation of our study was the difference in mode of delivery to the cancer experts (online) versus other participants (face-to-face) during the validation process. We designed the online tool so that experts were unable to return to previous pages to change responses. However, we recognize that experts could have searched the internet or consulted elsewhere before completing the tool online. We used Classical Test Theory to assess reliability and validity of the AWACAN tool. An alternative would be to use Item Response Theory [47,48]. However this would have required an extremely large sample size given the number of items in the questionnaire. Classical Test Theory was used in the development of the CAM and is adequate for this developmental phase, especially in a context with limited resources. The isiXhosa and Acholi versions of the AWACAN tool have recently being used in a cross-sectional study and further psychometric testing with these local language versions of the tool are being undertaken. In addition we intend to administer and assess validity of the AWACAN tool in other SSA countries. Important and interesting differences are likely to be identified with this broader administration.

We have demonstrated that the breast and cervical AWACAN tool has evidence of reliability and validity and is ready to be used as a measure of risk factor and symptom awareness, lay beliefs, help seeking behavior and barriers to care for the two leading causes of cancer morbidity among women in SSA.

## Supporting information

S1 Appendix The AWACAN tool for breast and cervical cancer English version. (PDF)

S2 Appendix Images of breast cancer changes. (PDF)
