# Supplementary material for: Development and validation of the African Women Awareness of CANcer (AWACAN) tool for breast and cervical cancer
Source: PLoS One. 2019 Aug 6;14(8):e0220545. doi: 10.1371/journal.pone.0220545 (PMC6684059; doi:10.1371/journal.pone.0220545)
Supplement: S1 Appendix — (PDF) [file pone.0220545.s001.pdf]

African Women Awareness of CANcer (AWACAN) breast and cervical cancer tool

English version

AWACAN
African Women Awareness of CANcer

SECTION 1: SOCIO-DEMOGRAPHIC QUESTIONS

No.	Questions	Response options
101.	How old are you?	___________ years
102.	What is your current relationship status?	Married
		Living together with a partner
		Single
		Separated/Divorced
		Widowed
		Did not answer
103.	What is your highest level of education?	No schooling
		Primary incomplete
		Primary complete
		Secondary incomplete
		Secondary complete
		More than secondary
		Did not answer
104.	What is the main language spoken at your home?	
105.	Do you have a job for which you get paid or from which you earn money?	Yes
		No
		Did not answer
106.	In what type of dwelling or housing do you live?	Brick house/apartment
		Informal dwelling/shack (e.g. in an informal or squatter settlement)
		Traditional dwelling/hut/structure made of traditional materials
		Other (please specify) ………………………………
		Did not answer

107.	Can you tell me whether you have any of the following where you live:	
	a) Do you have electricity or a generator or a solar panel?	Yes
		No
		Did not answer
	b) Do you have tap water in your house, compound, or property?	Yes
		No
		Did not answer
	c) Do you have any type of toilet in your house, compound, or property?	Yes
		No
		Did not answer
	d) Do you or does anyone living with you have a radio?	Yes
		No
		Did not answer
	e) Do you or does anyone living with you have a television?	Yes
		No
		Did not answer
	f) Do you or does anyone living with you have internet access on a computer, a laptop or a mobile phone?	Yes
		No
		Did not answer

SECTION 2: INTRODUCTION – BREAST CANCER SYMPTOM AWARENESS MEASURE

No.	Questions	Response options
201.	Have you ever heard of breast cancer?<br>**IF "NO" SKIP TO SECTION 402 = KNOWLEDGE OF SYMPTOMS**	Yes
		No
202.	Do you know of any family members, friends or neighbours who have/had breast cancer?	Yes
		No

SECTION 3: KNOWLEDGE OF RISK FACTORS

**301.** Please could you name as many things as you can think of that could increase **any** woman's chances of getting breast cancer?"

302. Could any of the following increase **any** woman's chances of getting breast cancer?"

No.	Questions	Response options
a)	Having had breast cancer previously	Yes
		No
		Don't know
b)	Drinking more than 1 bottle of beer or 1 glass of other types of alcohol per day	Yes
		No
		Don't know
c)	Not breastfeeding	Yes
		No
		Don't know
d)	Using hormone replacement therapy<br><br>*[Explanation]: As women get older their hormone levels become lower and their periods stop. They may experience hot flushes and other discomforts. The medication to help with these discomforts is called hormone replacement therapy*	Yes
		No
		Don't know
e)	Wearing a tight bra	Yes
		No
		Don't know
f)	Using hormonal family planning methods (e.g. the pill, injectable contraceptives and implants)	Yes
		No
		Don't know
g)	Being overweight	Yes
		No
		Don't know
h)	Wearing a bra all the time, including at night when sleeping	Yes
		No
		Don't know
i)	Having a family member with breast cancer	Yes
		No
		Don't know
j)	Putting money in one's bra	Yes
		No
		Don't know

k)	Having the first child after the age of 30 years	Yes
		No
		Don't know
l)	Bewitched/witchcraft/evil spirits	Yes
		No
		Don't know
m)	Starting your periods **early**, before the age of 11 years	Yes
		No
		Don't know
n)	Doing little physical activity or manual labour	Yes
		No
		Don't know
o)	Aging/growing old	Yes
		No
		Don't know
p)	Putting a mobile phone in one's bra	Yes
		No
		Don't know
q)	Having no children at all	Yes
		No
		Don't know
r)	Having menopause **late**, after the age of 55 years	Yes
		No
	[Explanation]: This is when a woman's period stops permanently	Don't know
s)	Being exposed to dirty air or water	Yes
		No
		Don't know

SECTION 4: KNOWLEDGE OF SYMPTOMS

**401.** Please would you name as many symptoms or signs of breast cancer as you can think of?"

402. Can you tell me if you think the following could be signs of something serious or that something is wrong, such as breast cancer?"

No.	Questions	Response options
a)	A change in the position of the nipple<br>*[Explanation]: such as pointing up or down or in a different direction to normal*<br>**(Picture available to illustrate)**	Yes<br>No<br>Don't know
b)	Pulling in of the nipple<br>*[Explanation]: Where the nipple no longer points outwards but into the breast*<br>**(Picture available to illustrate)**	Yes<br>No<br>Don't know
c)	A change in the size of the nipple, not when pregnant or breast feeding	Yes<br>No<br>Don't know
d)	A change in the shape of the nipple, not when pregnant or breast feeding	Yes<br>No<br>Don't know
e)	Nipple rash	Yes<br>No<br>Don't know
f)	Discharge from the nipple, not when pregnant or breast feeding	Yes<br>No<br>Don't know
g)	Bleeding from the nipple	Yes<br>No<br>Don't know
h)	Fever	Yes<br>No<br>Don't know
i)	Pain in one or both breasts, not when pregnant or menstruating	Yes<br>No<br>Don't know
j)	A lump or thickening in the breast	Yes<br>No<br>Don't know

k)	A change in colour of the breast skin, not when pregnant or breastfeeding	Yes
		No
		Don't know
l)	Puckering or dimpling of the breast skin<br>*[Explanation]: like a dent or orange peel appearance of the skin*<br>**(Picture available to illustrate)**	Yes
		No
		Don't know
m)	A change in the size of the breast, not when pregnant or breast feeding	Yes
		No
		Don't know
n)	A change in the shape of the breast, not when pregnant or breastfeeding	Yes
		No
		Don't know
o)	A lump or thickening under the armpit/under arm	Yes
		No
		Don't know
p)	Pain in the armpit/under arm	Yes
		No
		Don't know

SECTION 5: HELP-SEEKING BEHAVIOUR

No.	Questions	Response options
501.	a) If you noticed a change in your breast or breasts, would you ignore it?	Yes
		No
		Don't know
	b) If you noticed a change in your breast or breasts, would you try self-medication, for example get some ointment to apply from the local supermarket?	Yes
		No
		Don't know
	c) If you noticed a change in your breast or breasts, would you tell someone close to you?	Yes
		No
		Don't know

	d) If you noticed a change in your breast or breasts, would you visit a traditional healer?	Yes
		No
		Don’t know
	e) If you noticed a change in your breast or breasts, is there anything else you would do? (please specify):	
502.	**ONLY FOR THOSE WHO SAID ‘YES’ IN 501d** If you noticed a change in your breast or breasts, how soon would you visit a traditional healer?	< 1 week
		≥1 week < 1 month
		≥ 1 month < 3 months
		≥ 3 months
503.	**FOR ALL WOMEN**<br>If you noticed a change in your breast or breasts, how soon would you visit the pharmacy or clinic or health centre or hospital?	Never
		< 1 week
		≥1 week < 1 month
		≥ 1 month < 3 months
		≥ 3 months

SECTION 6: CONFIDENCE SKILLS AND BEHAVIOR IN RELATION TO BREAST CHANGES

No.	Questions	Response options
601.	Do you ever check your breasts?	Yes
		No
602.	Are you confident that you would notice a change in your breasts?	Yes
		No
		Don’t know
603.	Have you ever been to see a nurse or clinical officer or doctor about a change you have noticed in your breasts?	Yes
		No
		Never noticed a change in my breasts
604.	Have you ever been to see a traditional healer about a change you have noticed in your breasts?	Yes
		No
		Never noticed a change in my breasts

SECTION 7: INTRODUCTION – CERVICAL CANCER AWARENESS MEASURE

No.	Questions	Response options
701.	Have you ever heard of cervical cancer/cancer of the mouth/neck of the womb?<br><br>**IF "NO" SKIP TO SECTION 902 = KNOWLEDGE OF SYMPTOMS**	Yes
		No
702.	Do you know of any family members, friends or neighbours who have/had cervical cancer?	Yes
		No

SECTION 8: KNOWLEDGE OF RISK FACTORS

801. Please could you name as many things as you can think of that could increase **any** woman's chances of getting cervical cancer?"

802. Could any of the following increase **any** woman's chances of getting cervical cancer?"

No.	Questions	Response options
a)	Getting a sexually transmitted infection called the Human Papillomavirus (HPV)	Yes
		No
		Don't know
b)	HIV/AIDS	Yes
		No
		Don't know
c)	Being infected with other sexually transmitted diseases (other than HIV or Human Papillomavirus)	Yes
		No
		Don't know
d)	Using birth control pills/family planning for more than 5 years	Yes
		No
		Don't know

e)	Using condoms	Yes<br>No<br>Don't know
f)	Having unprotected sex	Yes<br>No<br>Don't know
g)	Smoking any cigarettes at all	Yes<br>No<br>Don't know
h)	Inserting herbs/creams/objects into the vagina	Yes<br>No<br>Don't know
i)	Having a sexual partner who is not circumcised	Yes<br>No<br>Don't know
j)	Having sex at a young age	Yes<br>No<br>Don't know
k)	Poor personal hygiene e.g. not washing one's vagina well, staying for long without bathing or wearing wet underpants	Yes<br>No<br>Don't know
l)	Giving birth to three or more children	Yes<br>No<br>Don't know
m)	Having many sexual partners	Yes<br>No<br>Don't know
n)	Not going for regular screening/testing for cervical cancer	Yes<br>No<br>Don't know
o)	Bewitched/witchcraft/evil spirits	Yes<br>No<br>Don't know

SECTION 9: KNOWLEDGE OF SYMPTOMS

901. Please could you name as many symptoms or signs of cervical cancer as you can think of?

902. Can you tell me if you think the following could be signs of something serious or that something is wrong such as cervical cancer?

No.	Questions	Response options
a)	Vaginal bleeding between menstrual periods	Yes
		No
		Don't know
b)	Persistent lower back pain	Yes
		No
		Don't know
c)	A persistent smelly vaginal discharge	Yes
		No
		Don't know
d)	Discomfort or pain during sex	Yes
		No
		Don't know
e)	Menstrual periods that are longer or heavier than usual	Yes
		No
		Don't know
f)	Persistent diarrhoea	Yes
		No
		Don't know
g)	Vaginal bleeding after menopause	Yes
	[Explanation]: Menopause is when a woman's periods have stopped permanently	No
		Don't know

h)	Persistent lower abdominal/pelvic pain	Yes<br>No<br>Don't know
i)	Vaginal bleeding during or after sex	Yes<br>No<br>Don't know
j)	Blood in urine or stool (faeces)<br><br>*[Explanation]: Blood in pee/wee or poo*	Yes<br>No<br>Don't know
k)	Unexplained weight loss	Yes<br>No<br>Don't know
l)	Itching in the vagina	Yes<br>No<br>Don't know

SECTION 10: HELP-SEEKING BEHAVIOUR

No.	Questions	Response options
	a) If you had a symptom coming from your cervix or mouth of your womb, would you ignore it?	Yes<br>No<br>Don't know
	b) If you had a symptom coming from your cervix or mouth of your womb, would you try self-medication, for example get some ointment to apply from the local supermarket?	Yes<br>No<br>Don't know
	c) If you had a symptom coming from your cervix or mouth of your womb, would you tell someone close to you?	Yes<br>No<br>Don't know
	d) If you had a symptom coming from your cervix or mouth of your womb, would you visit a traditional healer?	Yes<br>No<br>Don't know
	e) If you had a symptom coming from your cervix or mouth of your womb, is there anything else you would do? (please specify):	

1002.	**ONLY FOR THOSE WHO SAID 'YES' IN 1001d**<br>If you had a symptom coming from your cervix or womb, how soon would you visit a traditional healer?	< 1 week
		≥1 week < 1 month
		≥ 1 month < 3 months
		≥ 3 months
1003.	**FOR ALL WOMEN**<br>If you had a symptom coming from your cervix or womb, how soon would you visit the pharmacy/clinic/health centre/hospital?	Never
		< 1 week
		≥1 week < 1 month
		≥ 1 month < 3 months
		≥ 3 months

SECTION 11: CONFIDENCE SKILLS AND BEHAVIOR IN RELATION TO A CERVICAL CANCER SIGN/SYMPTOM

No.	Questions and filters	Response options
1101.	Are you confident that you would notice a symptom that could be cervical cancer?	Yes
		No
		Don't know
1102.	Have you ever been to see a nurse or clinical officer or doctor about a symptom that made you think something was wrong, like a symptom of cervical cancer?	Yes
		No
		Not noticed any symptoms or signs
1103.	Have you ever been to see a traditional healer about a symptom that made you think something was wrong, like a symptom of cervical cancer?	Yes
		No
		Not noticed any symptoms or signs

SECTION 12: BARRIERS TO SEEKING MEDICAL HELP [BREAST AND CERVICAL CANCER]

"Would any of the following reasons make it difficult for you to see the nurse or clinical officer or doctor if you noticed a symptom or sign which you think may be serious, for example a change in your breast or a change in the mouth of your womb or cervix that could be cancer?"

No.	Questions	Response options
a)	I would find it difficult to go for medical help because I would be **worried about wasting the nurse/clinical officer/doctor's time.**	Agree
		Disagree
		Don't know

b)	I would find it difficult to go for medical help because I would be **worried about what the nurse/clinical officer/doctor might find wrong.**	Agree<br>Disagree<br>Don't know
c)	I would find it difficult to go for medical help because I would be **worried about what tests the nurse/clinical officer/doctor might do.**	Agree<br>Disagree<br>Don't know
d)	I would find it difficult to go for medical help because I am **too busy or have other things to worry about.**	Agree<br>Disagree<br>Don't know
e)	I would find it difficult to go for medical help because **it takes too long to be seen at the clinic/health centre.**	Agree<br>Disagree<br>Don't know
f)	I would find it difficult to go for medical help because I have **no money for transport or the clinic/health centre charges.**	Agree<br>Disagree<br>Don't know
g)	I would find it difficult to go for medical help because I would **not feel confident about talking about my symptoms.**	Agree<br>Disagree<br>Don't know
h)	I would find it difficult to go for medical help because I have **had a bad experience in the clinic/health centre in the past.**	Agree<br>Disagree<br>Don't know
i)	I would find it difficult to go for medical help because I would **feel embarrassed.**	Agree<br>Disagree<br>Don't know
j)	I would find it difficult to go for medical help because the nurse/clinical officer/doctor would **not understand my language or culture.**	Agree<br>Disagree<br>Don't know
k)	I would find it difficult to go for medical help because **my husband/partner or family member would not allow me to go.**	Agree<br>Disagree<br>Don't know
l)	I would find it difficult to go for medical help because **I think that 'if I have a disease like cancer there is no use for the nurse/clinical officer/doctor and I will die anyway'.**	Agree<br>Disagree<br>Don't know
