# Supplementary material for: Development and validation of the African Women Awareness of CANcer (AWACAN) tool for breast and cervical cancer
Source: PLoS One. 2019 Aug 6;14(8):e0220545. doi: 10.1371/journal.pone.0220545 (PMC6684059; doi:10.1371/journal.pone.0220545)
Supplement: S2 Appendix — (PDF) [file pone.0220545.s002.pdf]

Image 1: Change in position of nipple

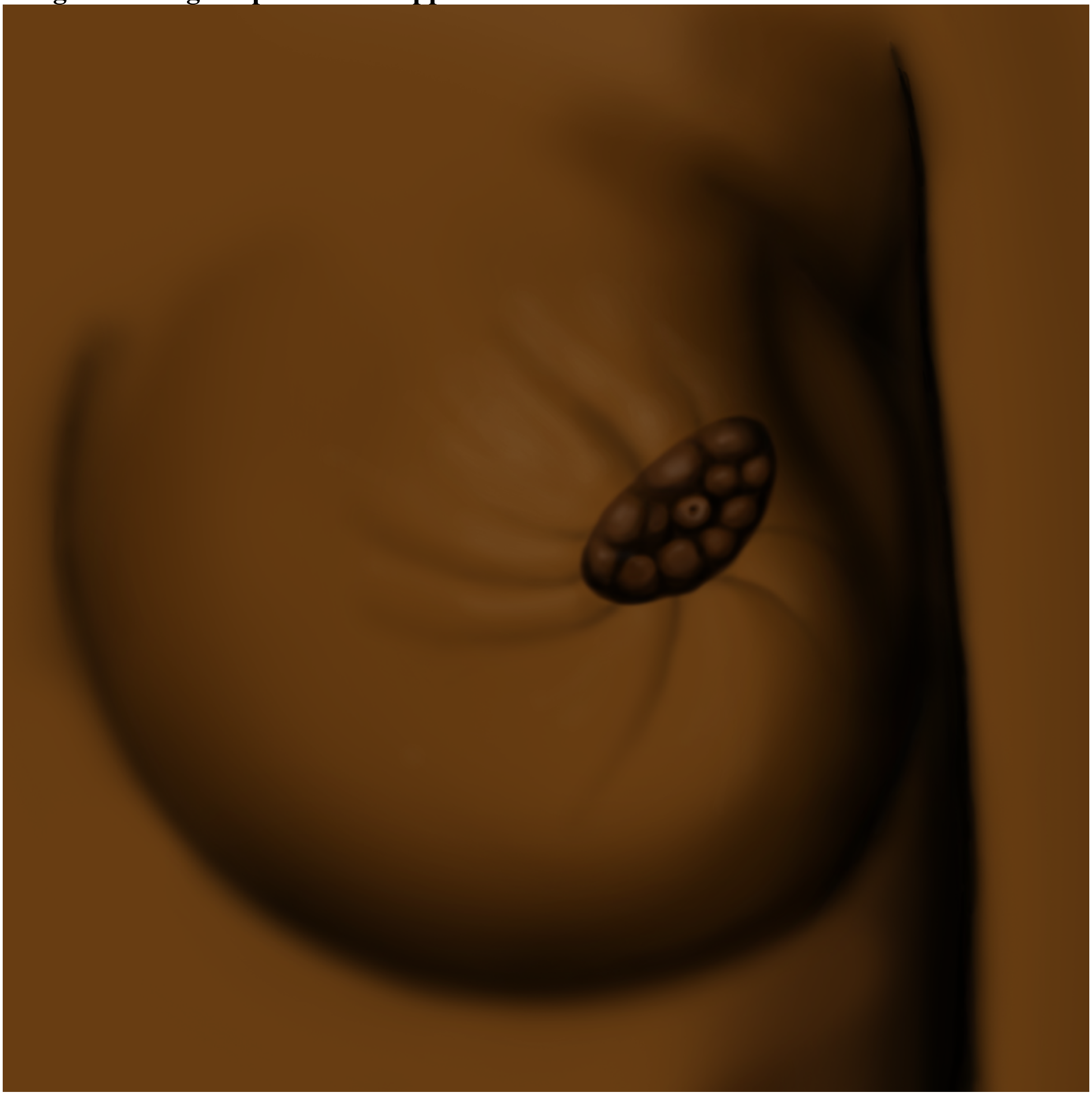

Images by Donovan Ward as commissioned by Dr Lydia Cairncross, Groote Schuur Hospital Breast Clinic, Cape Town, South Africa

Image 2: Nipple retraction

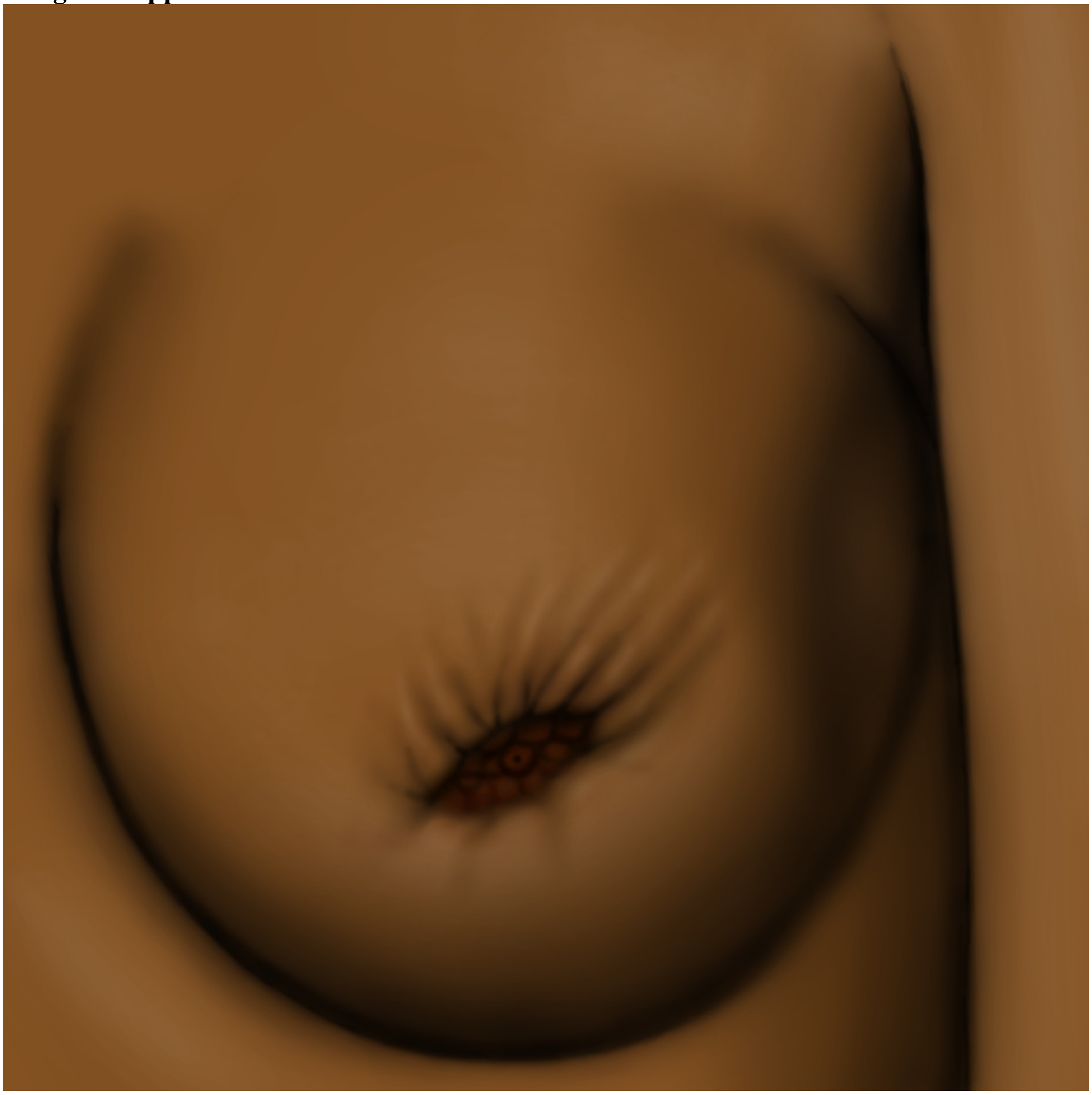

Images by Donovan Ward as commissioned by Dr Lydia Cairncross, Groote Schuur Hospital Breast Clinic, Cape Town, South Africa

Image 3: Orange peel appearance of skin

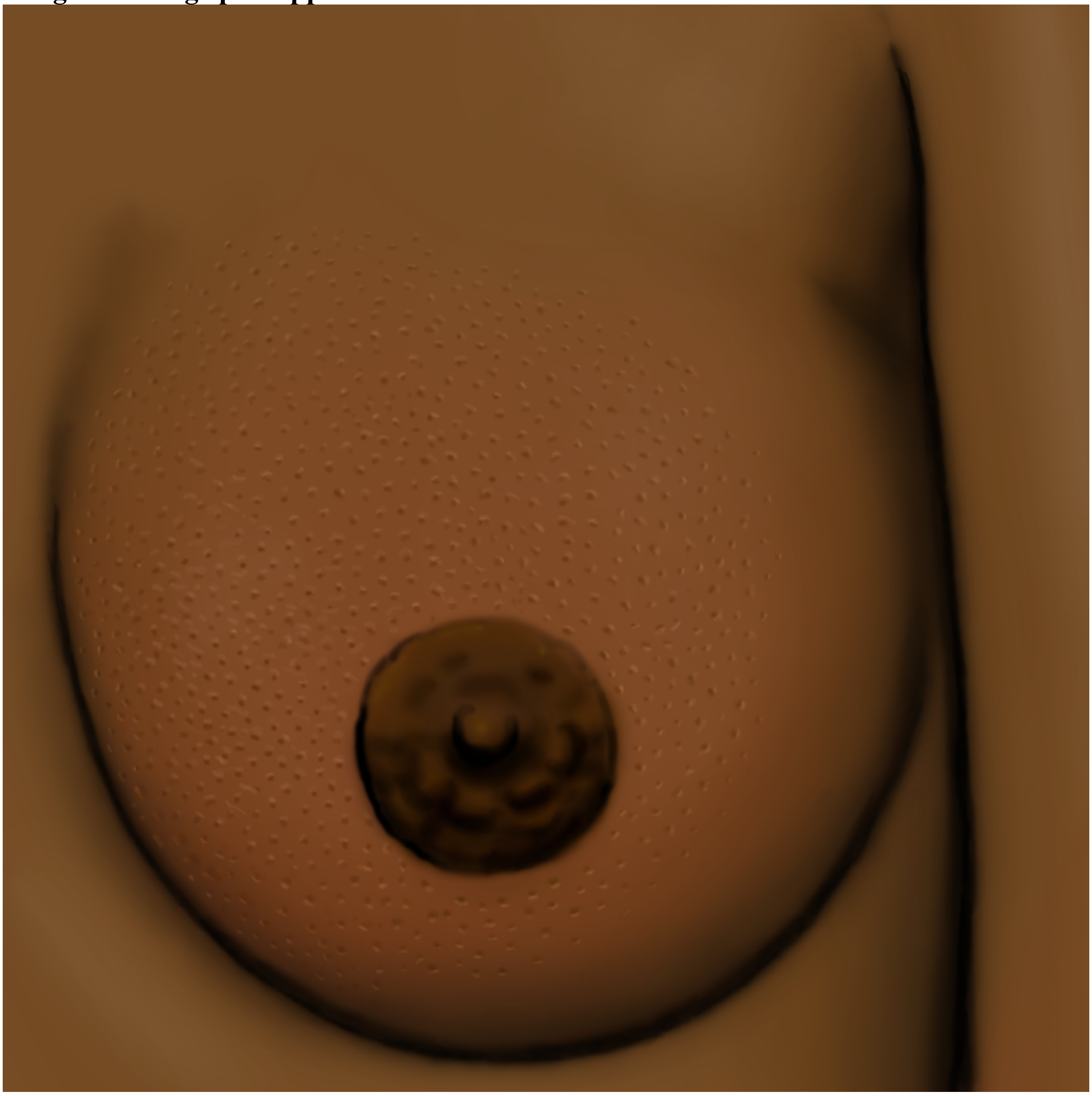

Images by Donovan Ward as commissioned by Dr Lydia Cairncross, Groote Schuur Hospital Breast Clinic, Cape Town, South Africa
